# APASL clinical practice guidance: the diagnosis and management of patients with primary biliary cholangitis

**DOI:** 10.1007/s12072-021-10276-6

**Published:** 2022-02-04

**Authors:** Hong You, Xiong Ma, Cumali Efe, Guiqiang Wang, Sook-Hyang Jeong, Kazumichi Abe, Weijia Duan, Sha Chen, Yuanyuan Kong, Dong Zhang, Lai Wei, Fu-Sheng Wang, Han-Chieh Lin, Jin Mo Yang, Tawesak Tanwandee, Rino A. Gani, Diana A. Payawal, Barjesh C. Sharma, Jinlin Hou, Osamu Yokosuka, A. Kadir Dokmeci, Darrell Crawford, Jia-Horng Kao, Teerha Piratvisuth, Dong Jin Suh, Laurentius A. Lesmana, Jose Sollano, George Lau, Shiv K. Sarin, Masao Omata, Atsushi Tanaka, Jidong Jia

**Affiliations:** 1 Liver Research Center, Beijing Friendship Hospital, Capital Medical University, 95 Yong-an Road, Beijing, Mainland, China; 2 Department of Gastroenterology and Hepatology, Renji Hospital, Shanghai Jiao Tong University, Shanghai, Mainland, China; 3 Department of Gastroenterology, Gazi Yaşargil Education and Research Hospital, Diyarbakir, Turkey; 4 Department of Infectious Diseases and Center for Liver Diseases, Peking University First Hospital, Beijing, Mainland, China; 5 Department of Internal Medicine, Seoul National University College of Medicine, Seoul National University Bundang Hospital, Seoul, South Korea; 6 Department of Gastroenterology, Fukushima Medical University School of Medicine, Fukushima, Japan; 7 Clinical Epidemiology and EBM Unit, Beijing Friendship Hospital, Capital Medical University, Beijing, Mainland, China; 8 Experimental and Translational Research Center, Beijing Clinical Research Institute, Beijing, Mainland, China; 9 Hepatobiliary Pancreatic Center, Tsinghua Changgung Hospital, Tsinghua University, Beijing, Mainland, China; 10 Treatment and Research Center for Infectious Diseases, The Fifth Medical Center of PLA General Hospial, Beijing, Mainland, China; 11 Division of Gastroenterology and Hepatology, Department of Medicine, Taipei Veterans General Hospital, Taipei, Taiwan; 12 Division of Hepatology, Department of Internal Medicine, College of Medicine, St. Vincent’s Hospital, The Catholic University of Korea, Suwon, South Korea; 13 Division of Gastroenterology, Department of Medicine, Faculty of Medicine, Siriraj Hospital, Mahidol University, Bangkok, Thailand; 14 Department of Internal Medicine, Cipto Mangunkusumo Hospital, University of Indonesia, Jakarta, Indonesia; 15 Department of Medicine, Fatima University Medical Center, Manila, Philippines; 16 Department of Gastroenterology, GB Pant Hospital, New Delhi, India; 17 Department of Infectious Disease and Hepatology Unit, Nanfang Hospital, Southern Medical University, Guangzhou, Mainland, China; 18 Department of Gastroenterology, Graduate School of Medicine, Chiba University, Chiba, Japan; 19 Department of Medicine, Ankara University School of Medicine, Ankara, Turkey; 20 School of Medicine, University of Queensland, Brisbane, Australia; 21 Department of Internal Medicine, National Taiwan University Hospital, Taipei, Taiwan; 22 NKC Institute of Gastroenterology and Hepatology, Faculty of Medicine, Prince of Songkla University, Hatyai, Thailand; 23 Department of Gastroenterology, University of Ulsan College of Medicine, Seoul, South Korea; 24 Digestive Disease and GI Oncology Centre, Medistra Hospital, Jakarta, Indonesia; 25 Department of Medicine, University of Santo Tomas, Manila, Philippines; 26 Humanity and Health Clinical Trial Center, Humanity and Health Medical Group, Hong Kong SAR, China; 27 Department of Hepatology, Institute of Liver and Biliary Sciences, Vasant Kunj, New Delhi, India; 28 Department of Gastroenterology, Yamanashi Central Hospital, Yamanashi, Japan; 29 Department of Medicine, Teikyo University School of Medicine, Tokyo, Japan; 30 University of Tokyo, Tokyo, Japan

## Introduction

Primary biliary cholangitis (PBC) is a chronic intrahepatic cholestatic disease with not fully elucidated pathogenesis. Immunological dysfunction triggered by environmental factors may render autoimmunity against the interlobular bile ducts in genetically predisposed hosts. PBC typically affects middle-aged women, commonly presents with fatigue and pruritus, or with an asymptomatic elevation of serum alkaline phosphatase (ALP)/glutamyl transpeptidase (GGT). The pathological features are progressive, non-suppurative, destructive intrahepatic cholangitis, leading to fibrosis and eventually cirrhosis. Antimitochondrial antibodies (AMAs), especially the M2 subtype (AMA-M2), are highly sensitive and specific for PBC in clinical settings. Currently, ursodeoxycholic acid (UDCA) is the treatment of choice for this disease.

In response to the increasing report of this once-regarded rare disease of the Western world in the Asia–Pacific region, a panel of invited expert hepatologists and methodologists developed the Asian Pacific Association for the Study of the Liver (APASL) Clinical Practice Guidance on the Diagnosis and Management of Patients with Primary Biliary Cholangitis.

## Guidance development process

The invited panel of clinicians with expertise in PBC and methodologists with special interest in clinical research of liver diseases drafted and discussed this guidance. We conducted a formal literature review of evidence from PubMed and Cochrane database as of January 2021. In developing recommendations and supporting texts, the expert methodologists assisted in assessing the quality of identified evidence using the Grading of Recommendations Assessment Development and Evaluation (GRADE system) [1] (Table 1).

**Table 1:** Grading evidence and recommendations

Grade of evidence	
I	High quality: Further research is very unlikely to change our confidence in the estimate of effect
II	Moderate quality: Further research is likely to have an important impact on our confidence in the estimate of effect and may change the estimate
III	Low quality: Further research is very likely to have an important impact on our confidence in the estimate of effect and is likely to change the estimate
IV	Very low quality: Any estimate of effect is very uncertain
Grade of recommendation	
1	Strong recommendation: recommendation is made on the consideration of benefit, patients’ wishes, cost and resources
2	Weak recommendation: recommendation is made with less certainty, higher cost or resource consumption

## Epidemiology

PBC may affect all races and ethnicities with great geographical variation [2, 3]. Overall, the estimated global incidence and prevalence were 17.6 per million persons/year and 146 per million, respectively [3]. The reported incidence and prevalence of PBC in the Asia–Pacific region (8.4, and 98.2–118.8 per million, respectively) were lower than that in North America (27.5 and 218.1 per million, respectively) and Europe (18.6 and 145.9 per million, respectively) [3, 4]. Of note, the geographical differences in PBC epidemiology exist even within the Asia–Pacific region,with a higher reported prevalence in Japan and China (191.18 per million) and a much lower reported prevalence in South Korea and Australia (39.09 per million) [4]. Interestingly, the reported prevalence of PBC in New Zealand and Australia is much lower than that in Europe despite the fact that their populations share similar genetic background, adding further weight to the hypothesis that environmental factors may play a role in the etiopathogeneis of PBC [5, 6].

The prevalence of PBC in the Asia–Pacific region has become higher than once deemed and increased quickly [3, 7–14]. A recent study in Japan demonstrated that the point prevalence of PBC was 338 per million, which was comparable to that in Europe and North America [12]. Another Japanese study reported that PBC was diagnosed in 5.7% of the women with asymptomatic serum GGT elevation (6.0% among all the women) at the annual health check-up among a large population, yielding an estimated PBC prevalence of 3400 per million in women over 40 years old and 840 per million in the whole population in Okinawa Prefecture [7].

## Pathogenesis

The interplay of environmental, genetic/epigenetic, and immunological factors play a crucial role [15, 16], although the exact pathogenesis of PBC remains elusive. Environmental factors, such as cigarette smoking [17], toxin exposure [18], and infectious agents [19], may breakdown the immune tolerance in individuals with genetic susceptibility. It has been reported that infected microbes could act as cross-antigens and cause molecular mimicry, thereby breaching the self-tolerance and initiating autoimmune reactions against intrahepatic bile ducts [15]. Meanwhile, gut dysbiosis and geographical clustering of PBC cases indicate that gut microbiota and environmental influence may be potential risk factors for the disease [20, 21].

Familial and genetic studies highlight the importance of genetic susceptibility for PBC. Recently, genome-wide association studies (GWAS) have identified multiple genes conferring PBC susceptibility in human leucocyte antigen (HLA) and non-HLA loci [22, 23]. Studies have shown that HLA DRB1*11 and HLA-DRB1*13 are protective against PBC in European cohorts, whereas HLA-DQB1*06:04 and DQB1*03:01 are protective against the disease in Japanese cohorts [16]. In Chinese Han, HLA-DQB1*03:01 confers PBC resistance, whereas HLA-DRB1*08:03 and HLA-DPB1*17:01 confer PBC susceptibility [16]. Moreover, it is reported that HLA-DRB1*03:01 was significantly associated with anti-sp100 positive subphenotype of PBC in Chinese Han [24].

One of the significant non-HLA genes revealed by the GWAS is the interleukin-12 (IL-12) pathway, which may participate in developing auto-reactive Th1 cells, thereby rendering the PBC onset [22, 25]. Recently, a Japanese study on meta-analysis of GWAS and bioinformatics demonstrated that protein O-glucosyltransferase 1 (POGLUT1) is the effector gene regulated by the primary functional SNP rs2293370 (a susceptibility locus for PBC on chromosome 3q13.33) [26]. In turn, higher endogenous levels of POGLUT1 may induce excessive Notch signaling, thereby mounting immune responses against self-antigens [26]. Genetic studies in Chinese Han population have not only confirmed associations of several risk loci previously found in Europeans or Japanese, but also identified novel risk factors for PBC, such as desregulation of IL-21 signaling pathway [27]. Notably, known risk variants merely account for less than 20% heritability of PBC, indicating that other factors contribute to their genetic background.

The innate immunity is implicated in the pathogenesis of PBC, as indicated by the presence of granulomatous inflammation, the hypersecretion of proinflammatory cytokines and polyclonal immunoglobulin M (IgM), the elevation of NK and NKT cells, as well as noticeable hyperresponsiveness to CpG oligodeoxynucleotides [28, 29]. Pathogen-associated molecular patterns (PAMP) can bind to toll-like receptors (TLRs) on the surface of biliary epithelial cells (BECs) and innate immune cells, thereby triggering innate immunity [30]. Meanwhile, monocytes, activated by the PAMP through TLRs, participate in the modulation or amplification of adaptive cellular immune response by secreting proinflammatory cytokines (e.g., IL-1, IL-6, IL-12, and TNF-α) [31]. During PBC progression, abnormaly retented bile acids can signal through various nuclear receptors, thereby regulating immune responses [32].

The adaptive immunity also participates in the pathogenesis of PBC, as indicated by the presence of a high concentration of antimitochondrial antibodies specific for 2-oxo-acid dehydrogenase complex (2-OADC) and the increase of antigen-specific CD4+ and CD8+ T cells [25]. CD8+ T cells are the predominant infiltrating lymphocytes in the liver tissues of PBC patients, which express FasL and secret perforin thereby leading to apoptosis of BECs [33]. Regulatory T lymphocytes (Treg), which suppress self-reactive CD8+ lymphocytes and regulate inappropriate immune responses, are significantly lower in PBC patients and their family members [34]. This finding suggests that dysfunction of the Treg cells may reduce immune tolerance and confer effector lymphocytes to damage the BEC. Besides, myeloid-derived suppressor cells [35], double-negative T cells (DNT) [36], and mucosal-associated invariant T cell (MAIT) [37] were also implicated in the development of PBC. However, the exact roles of these cells are still not fully elucidated.

Intriguingly, injured BECs of PBC patients can express higher level of HLA class II molecules and act as non-professional antigen-presenting cells. The interplay of BECs and T cells may, to some extent, account for bile duct loss, a key characteristic of disease progression [25]. Additonally, the bone marrow (BM) microenvironment might also play a role in the pathogenesis of PBC. The hemopoietic progenitor cells and stromal cells were defective [38], and the BM cytokines and apoptotic process were altered in PBC [39, 40].

**Fig. 1:**
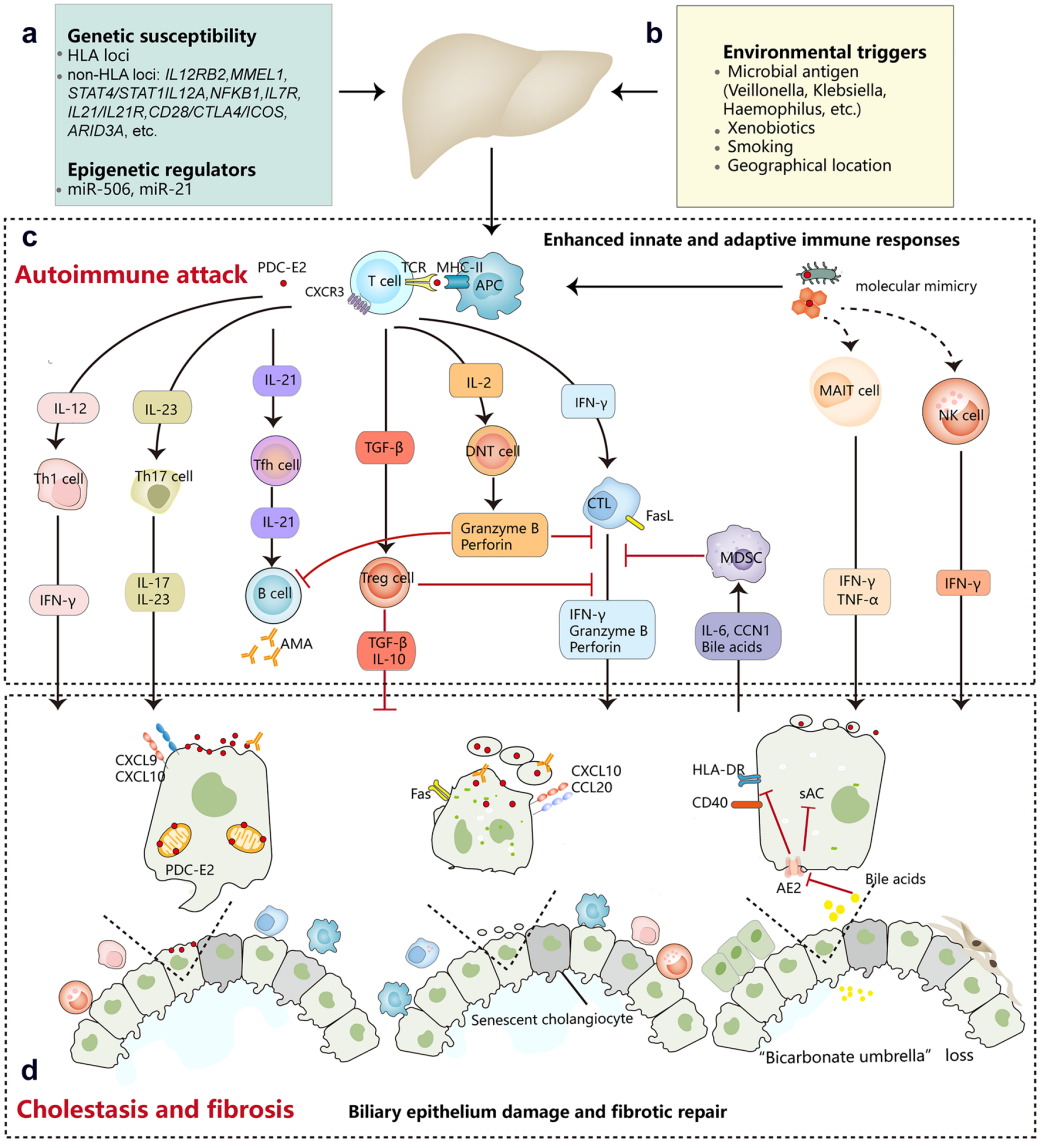
Pathogenesis of primary biliary cholangitis. PBC is complex and is thought to be caused by the interplay of genetic (**A**) and environmental factors (**B**, **C**). Exposure to PDC-E2 initiates innate and adaptive immune responses that target biliary epithelial cells and cause inflammation. **D** Injured cholangiocytes with dysfunctional anion exchanger 2 (AE2) are sensitive to apoptosis and senescence, ultimately leading to cholestasis and liver fibrosis. *HLA* human leucocyte antigen, *miR-506* microRNA 506, *miR-21* microRNA 21, *PDC-E2* the E2 component of the mitochondrial pyruvate dehydrogenase complex, *CXCR3* C-X-C motif chemokine receptor 3, *APC* antigen presenting cell, *IFN-γ* interferon-γ, *Tfh* follicular helper T cell, *AMA* anti-mitochondrial autoantibody, *TGF-β* transforming growth factor-β, *Treg* regulatory T cells, *DNT* double negative T cell, *CTL* cytotoxic T lymphocyte, *FasL *Fas ligand,  *MDSC* myeloid-derived suppressor cell, *CCN1* cellular communication network factor 1, *MAIT* mucosal-associated invariant T cell, *TNF-α* tumor necrosis factor-α, *NK* natural killer, *CXCL9* C-X-C motif chemokine ligand 9, *CXCL10* C-X-C motif chemokine ligand 10, *CCL20* C-C motif chemokine ligand 20, *sAC* soluble adenylyl cyclase, *AE2* anion exchanger 2

Putting together, the interplay of environmental and immunological factors in an individual with genetic susceptibility breaches the autotolerance to and mounts autoimmunity against the intrahepatic BECs, thereby leading to the characteristic pathological and clinical phenotypes of PBC (Fig. 1).

## Diagnosis of PBC

### Clinical features

PBC typically affects middle-aged women, with a female to male ratio as high as 10:1 [41–44]. However, the female to male ratio (3.9–6.2:1) was much lower in recently reported cohorts from Korea [11], Japan [12], China Mainland [45], Hong Kong [13] and Taiwan [46].

The clinical manifestation of PBC includes pruritus, fatigue, and, less commonly, jaundice or complications of cirrhosis. Nowadays, an increasing number of asymptomatic patients are diagnosed at an early disease stage mainly due to routine testing of liver biochemistry [47], especially in East Asia. It is reported that the mortality of the elderly asymptomatic PBC patients (≥ 55 years) were similar to that of the age- and sex-matched general population [48]. However, if left undiagnosed or untreated, two thirds of the asymptomatic patients will eventually develop into the symptomatic phase within five years [49].

#### Common symptoms

Symptomatic PBC patients usually present with fatigue, pruritus, or jaundice. Fatigue occurs in up to 80% of patients and fluctuates independently of disease activity or stage [50]. The pathogenesis of fatigue is not entirely clear but central nervous system abnormalities caused by cholestasis and peripheral muscle dysfunction have been implicated, which lead to autonomic dysfunction, daytime sleepiness, night sleep disturbance, impaired concentration, memory problems and depression [51]. The Fatigue Impact Scale (FIS) [52], especially the PBC-40 [53] helps to measure the severity of fatigue in PBC patients [51]. Early presentation of fatigue severely affects the health-related quality of life (HRQoL) and has been viewed as a predictor of mortality. Furthermore, fatigue may not be completely resolved by UDCA or even liver transplantation [54].

Pruritus affects 20–70% of patients, making it another frequent complaint in PBC patients [55]. A recent cross-sectional study in Japan showed that about 30% of patients with PBC suffered from moderate-to-severe pruritus [56], which is annoying very much but may not be noticed as a manifestation of PBC. Pruritus in PBC is thought to be mediated by pruritogens, such as bile salts, autotaxin, and lysophosphatidic acid, which are normally excreted into the bile but accumulates in the serum as a result of cholestasis [57]. It may occur in any stage, before, at, or after the development of jaundice. Pruritus is usually mild and tolerable in most PBC patients, but it may be severe and persistent in some patients, thus compromise the HRQoL. However, the severity of pruritus seems to not correlate with the disease stage or activity.

#### Associated diseases and syndromes

Several extrahepatic autoimmune diseases could coexist with PBC, such as Sjögren’s syndrome (3.5–73%), autoimmune thyroid disease (5.6–23.6%), systemic sclerosis (1.4–12.3%), Raynaud’s phenomenon (1.8–5.6%), systemic lupus erythematosus (0–3.7%), and celiac disease (0–6%) [58]. Other less recognized diseases with a relatively lower prevalence could also occur in PBC patients. However, these associated syndromes do not change the natural history, clinical presentation, or survival of PBC [59, 60].

Studies found that specific autoantibodies of rheumatologic disorders including antibodies against SS-A/Ro-52kD and centromere might be associated with the diagnosis and poorer prognosis of PBC [61, 62]. Obviously, the significance of these autoantibodies needs to be further explored.

#### Complications

If left untreated, PBC patients with persistent cholestasis will eventually progress to the advanced stage with complications associated with cholestasis and/or cirrhosis.

Hyperlipidemia, which results from complex processes of biliary cholestasis, is common in PBC patients. It can cause xanthelasmas and xanthomas due to the remarkable elevation of high-density lipoprotein cholesterol. Interestingly, this kind of hyperlipidemia seems not to confer an increased risk of cardiovascular disease [63].

Compared with age-matched healthy people, PBC patients are more inclined to have hepatic osteodystrophy, such as osteoporosis which affects around 20–44% of the patients [64]. Fat-soluble vitamin malabsorption may occur due to decreasing secretion in bile acid, but a significant lack of vitamin A, D, E, and K are uncommon [65].

Complications associated with cirrhosis and portal hypertension such as ascites, gastroesophageal variceal bleeding, hepatic encephalopathy seem to resemble those caused by other chronic liver diseases. Of note is that signs of portal hypertension can develop even before the establishment of cirrhosis, which is presinusoidal in nature [66]. The risk of hepatocellular carcinoma (HCC) in PBC patients also increased, especially in men or those who have already developed cirrhosis [67–69].

### Laboratory tests

#### Biochemical tests

**Fig. 2:**
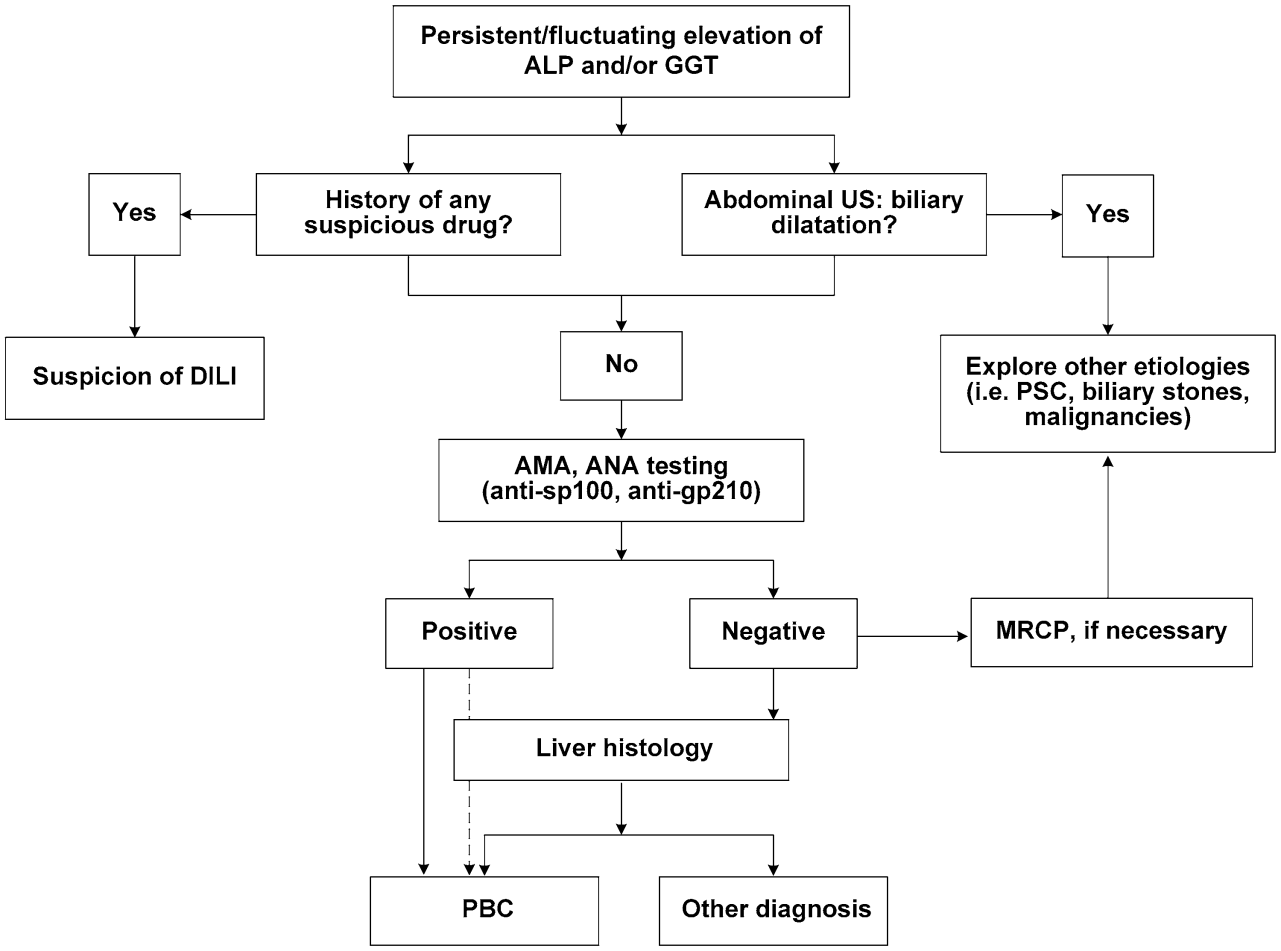
Diagnostic flowchart for PBC. *PBC* primary biliary cholangitis, *ALP* alkaline phosphatase, *GGT* gamma glutamyl transferase, *US* ultrasonography, *PSC* primary sclerosing cholangitis, *AMA* anti-mitochondrial autoantibody, *DILI* drug induced liver injury

PBC patients may have abnormal biochemical tests, such as increased ALP and GGT, mild elevation of aminotransferases, and elevation of immunoglobulins (mainly IgM). Figure 2 depicts a diagnostic workup for patients with elevated cholestatic enzymes.

#### Immunological tests

##### Anti-mitochondrial antibody (AMA)

AMA, particularly the AMA-M2 subtype, is a serological diagnostic hallmark for PBC, with sensitivity and specificity being > 90–95% [70, 71]. AMAs recognize 2-oxo acid dehydrogenase complex(2-OADC) located at the inner membrane of the mitochondria, which mainly includes the E2 component of the pyruvate dehydrogenase complex (PDC-E2), branched-chain 2-OADC (BCOADC-E2), 2-oxo-glutaric acid dehydrogenase complex (OGDC-E2), and dihydrolipoamide dehydrogenase binding protein (E3BP). Of note is that the serum level of AMA does not reflect the disease severity of PBC [72].

In clinical practice three methods were commonly used to detect AMA, including immunofluorescence (IIF), enzyme-linked immunosorbent assay (ELISA) and Western immunoblot. IIF on fresh frozen rodent kidney, stomach and liver tissues is considered as the initial routine screening [73]. AMA testing with ELISA (AMA-M2), which utilizes all three major autoantigens of AMA(recombinant PDC-E2, BCOADC-E2, and OGDC-E2 proteins) has a higher specificity and sensitivity than IIF [74, 75]. For those with clinical suspicion of PBC but negative results by IIF and ELISAs, complementary testing with Western immunoblot against mitochondrial antigens could be of value for diagnosis as Western blotting is very specific for AMA detection [76]. Of note, AMA can occasionally be detected in non-PBC subjects, including autoimmune hepatitis (AIH), systemic lupus erythematosus, Sjogren syndrome, chronic hepatitis C, chronic bacterial infection, or even healthy persons, or transiently positive among patients with acute liver failure of any etiology [77].

##### Anti-nuclear autoantibodies

Anti-nuclear autoantibodies (ANAs) are important ancillary diagnostic markers of PBC, which are detectable by IIF in up to 50% of PBC patients [70]. Certain ANA patterns are highly specific for PBC, including the rim-like membranous pattern targeting gp210 and p62, and the multiple nuclear dots pattern targeting several proteins including sp100. Meta-analysis showed that anti-gp210 and anti-sp100 have a low sensitivity (15 ~ 40%), but a high specificity (bother greater than 95%) for AMA-negative PBC [78]. In a large study with more than 4000 tested sera, simultaneous positivity for both anti-sp100 and anti-gp210 has shown a 100% positive predictive value for PBC irrespective of the AMA status [79].

Recently, antibodies against two novel PBC autoantigens, kelch-like 12 (KLHL12) and hexokinase 1, have been found in 35% and 22% of AMA-negative PBC patients, respectively [80]. In addition, anti-promyelocytic leukemia protein (anti-PML), anti-SUMO, anti-Sp140, anti-lamin B receptor and anticentromere all have been found in PBC patients, but their clinical significance remains elusive [81–84].

### Imaging examinations

Individuals with cholestasis should routinely be examined with ultrasonography. For those with intrahepatic or extrahepatic bile duct dilation on ultrasonography, a diagnosis of PBC is very unlikely. Instead, magnetic resonance cholangiopancreatography (MRCP), endoscopic retrograde cholangiopancreatography (ERCP), or endoscopic ultrasonography should be considered to rule out other biliary diseases, including cholelithiasis, inflammation (such as primary sclerosing cholangitis, PSC) or manlignacy [85].

Noninvasive techniques, such as transient elastography (TE) or magnetic resonance elastography (MRE), have been evaluated for staging PBC [86, 87]. TE has also been used in longitudinally monitoring the progression of PBC patients [86].

### Pathological characteristics and histological staging

Macroscopically, the liver is enlarged in the early stages and can be bile stained. The cirrhotic liver of PBC is generally larger than cirrhosis of other etiology such as viral hepatitis or AIH.

**Fig. 3:**
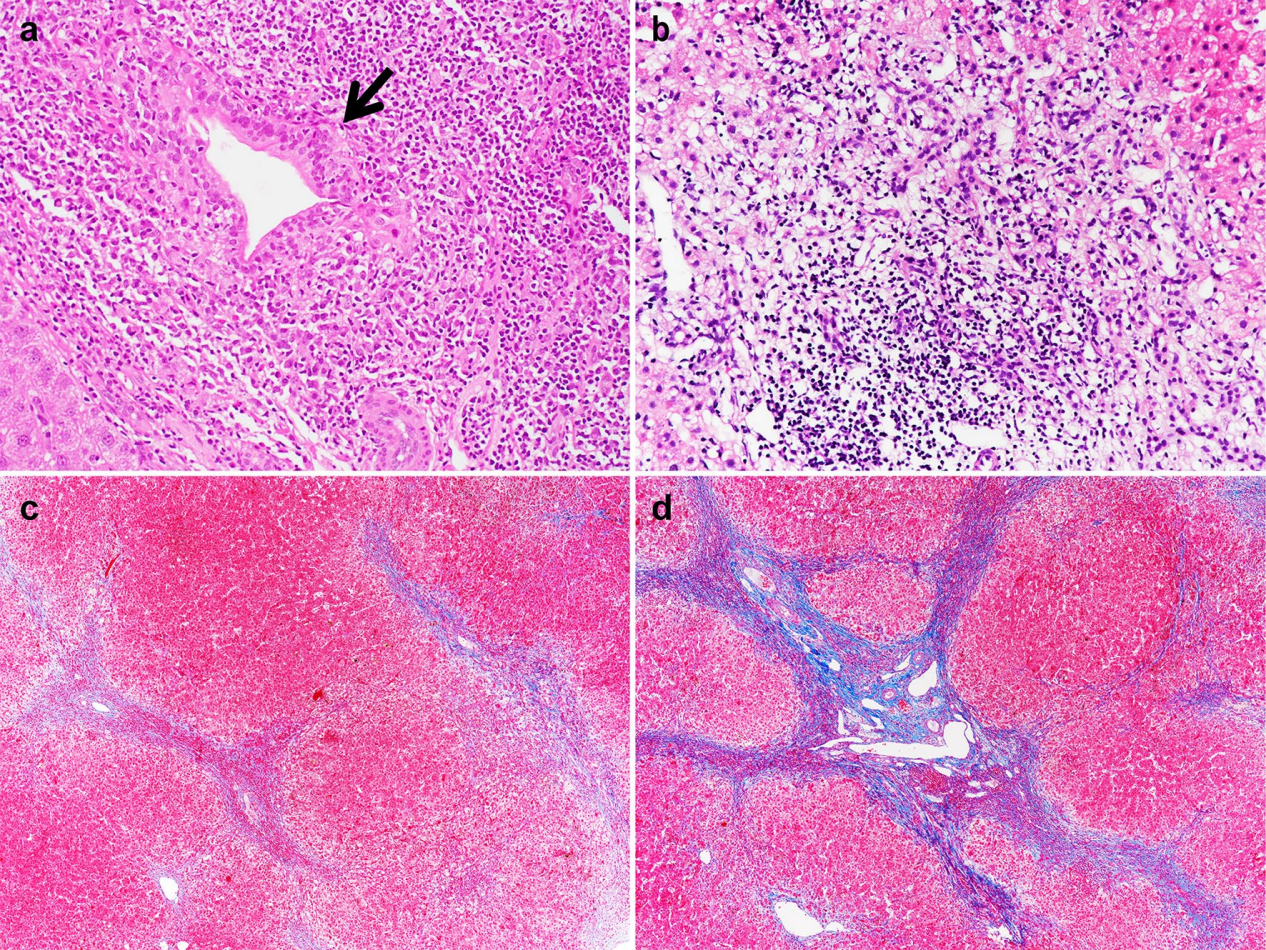
Typical histological features of PBC in different stages. **a** Stage I: chronic non-suppurative destructive cholangitis (arrow, H&E, 200×). **b** Stage II: ductular reaction with periportal necroinflammotory activity (H&E, 200×). **c** Stage III: multiple portal-portal bridging fibrosis (Trichrome, 40×). **d** Stage IV: biliary cirrhosis with nodule formation (Trichrome, 40×)

Histologically, PBC is characterized by chronic, nonsuppurative cholangitis that mainly affects interlobular and septal bile ducts (Fig. 3). Focal lesions showing intense inflammatory infiltration and necrosis around bile ducts are termed as “florid duct lesion” and almost pathognomonic for PBC [88, 89]. The inflammatory infiltration consists of lymphocytes and other mononuclear cells in close contact with the basal membrane of cholangiocytes undergoing necrosis. Bile duct paucity or ductopenia is usually defined as less than 50% of portal tracts containing bile ducts.

Histologic lesions of PBC are classically divided into four stages [88, 89], according to the degree of bile duct damage, inflammation, and fibrosis. Stage I is characterized by portal inflammation with or without florid bile duct lesions. Epithelioid granulomas are present in some cases, usually in stage I or II. Stage II is characterized by periportal lesions extending into the hepatic parenchyma; the severity of lymphocytic or biliary interface hepatitis is highly predictive of extensive fibrosis development [90, 91]. Stage III is characterized by a distortion of the hepatic architecture with numerous fibrous septa. Stage IV is defined as cirrhosis with the existence of regenerative nodules.

Recently, Nakanuma et al. proposed new histological assessment criteria for PBC, which consist of both grading (cholangitis activity and hepatitis activity) and staging (scoring of fibrosis, bile duct loss, and deposition of orcein-positive granules) [92]. The novel criteria for histology could stratify the risk for PBC progression and outcomes [93, 94]. More recently, PML expression was found highly specific for histological diagnosis of PBC; therefore, it could be used to discriminate PBC from other liver diseases including small-duct PSC [95].

### Diagnosis criteria

The diagnosis of PBC is based on the results of biochemical, immunological, radiological, and histological investigations.

*Recommendations*:

1. The diagnosis of PBC can be established when meeting two or more of the following three criteria: (I, 1)

1. Biochemical evidence of cholestasis based mainly on the elevation of ALP and GGT with the exclusion of extrahepatic biliary obstruction by imaging studies;
2. Presence of AMA or other PBC-specific ANAs including anti-sp100 or anti-gp210;
3. Histologic evidence of non-suppurative destructive cholangitis mainly affecting the interlobular bile ducts.

### Differential diagnosis

The differential diagnosis of PBC includes extrahepatic or intrahepatic cholestasis of any other causes (Table 2). Of note is that PSC is characterized by multi-focal bile duct strictures, which can involve intrahepatic, extrahepatic bile ducts, or both. Radiologically mimicking PSC, IgG4-associated sclerosing cholangitis is characterized by high levels of IgG4 in serum along with dense infiltration of IgG4-positive lymphoplasma cells in the tissue.

**Table 2:** Differential diagnosis of PBC

Intrahepatic cholestasis	Extrahepatic cholestasis
Hepatocyte-associated Autoimmune hepatitis Alcoholic liver disease Drug-induced liver disease Bile duct-associated Primary sclerosing cholangitis Secondary sclerosing cholangitis IgG4-associated sclerosing cholangitis Vascular diseases Sinusoidal obstruction syndrome Budd-Chiari syndrome Congestive hepatopathy Miscellaneous Sarcoidosis Hepatic amyloidosis Langerhans cell histiocytosis	Cholelithiasis Inflammatory stenosis Malignancy

Langerhans cell histiocytosis (LCH) is a rare systemic disorder characterized by wide-ranging organ involvement and the accumulation of CD1a+ /Langerin + LCH cells in the tissue.

Sarcoidosis is manifested by non-caseating granulomas within involved organs, most commonly the pulmonary, lymphatic, and hepatic systems.

Amyloidosis is a rare disease that may involve the kidney, heart, liver, and other organs. The clinical manifestations of hepatic involvement by amyloidosis are usually mild, including hepatomegaly and elevation of ALP, but hepatic failure and portal hypertension may develop in some cases.

## Management of PBC

### Lifestyle modification

The possible role of smoking has been implicated in the development and progression of PBC [96]. The alcohol intake (> 20 g/d) was usually associated with smoking history and may also contribute to the disease progression [96]. In one study, non-alcoholic steatohepatitis (NASH) and body mass index ≥ 25 were also associated with severer biliary duct damage and fibrosis in PBC patients, but another study did not confirm this result [97, 98]. In general, lifestyle modifications such as smoking cessation, alcohol abstinence, and body weight reduction would be justified in PBC patients to improve the clinical outcomes.

*Recommendation*:

2. In the context of the negative impacts of cigarette smoking, alcohol intake, and obesity on human health, PBC patients should be encouraged to quit smoking, stop alcohol drinking, and keep on ideal body weight. (III, 2).

### First-line treatment: UDCA

UDCA is the treatment of choice and most established therapy for PBC patients, recommended by major national and international clinical practice guidelines [42–44, 99, 100]. Its primary mode of action is to exert the choleretic effect and protect hepatocytes and cholangiocytes against the cytotoxicity of hydrophobic bile acids. The optimal dosage of UDCA is 13 ~ 15 mg/kg per day in a single or divided oral doses. Studies show that low-dose UDCA (5 ~ 7 mg/kg per day) is less effective, but high-dose UDCA (23 ~ 25 mg/kg per day) does not bring more benefits [101].

Tauroursodeoxycholic acid (TUDCA) is a taurine conjugated form of UDCA with higher hydrophilicity. TUDCA 750 mg/day showed similar biochemical efficacy and safety profile to UDCA 750 mg/day in a multicenter randomized clinical study [102].

Many randomized controlled trials and meta-analyses demonstrated that UDCA can improve biochemical variables [103–105], halt the disease progression [106, 107] and prolong the liver transplant-free survival [108, 109]. UDCA is safe and well-tolerated in PBC patients. The frequently reported side effects are diarrhea, flatulence, weight gain, and pruritus aggravation. These side effects are generally minor and do not require therapy withdrawal. Despite its excellent safety profile, a recent study showed 11% of the UDCA-treated patients showed poor adherence to the therapy [110]. Young age and male sex were independently associated with poor adherence.

*Recommendation*:

3. It is recommended that oral UDCA (13 ~ 15 mg/kg/day) should be standard therapy for all PBC patients. UDCA treatment should be continued for prolonged periods, and compliance to therapy should be checked (I, 1).

### Criteria of biochemical response to UDCA

Unfortunately, about 30–40% of PBC patients show insufficient biochemical responses to UDCA and remain at risk for disease progression to advanced stages, including cirrhosis [111–114]. Several criteria of biochemical response were proposed to help the risk stratification of PBC patients and to identify those who need second-line therapy (Table 3) [111, 115–121], which were summarized in a recent review [122]. Among many, Paris I [111] and Paris II criteria [121] are well-validated and widely used criteria of biochemial response in patients with advanced PBC (stage III-IV) and early PBC (stage I–II), respectively.

**Table 3:** Evaluation of response to UDCA therapy in patients with PBC

UDCA-response criteria	Time (months)	Definition of response
Barcelona [115]	12	> 40% decrease or normalization of ALP
Mayo [116]	6	ALP < 2 × ULN
Paris I [111]	12	ALP ≤ 3.0 × ULN and AST ≤ 2.0 × ULN and normalization of bilirubin
Rotterdam [117]	12	Normalization of abnormal bilirubin and/or albumin
Ehime [118]	6	≥ 70% decrease or normalization of GGT
Toronto [119]	24	ALP ≤ 1.67 × ULN
Paris II [121]	12	ALP and AST ≤ 1.5 × ULN and normalization of bilirubin
Risk scoring systems		Included parameters
GLOBE [123]	12	Age at diagnosis. ALP, bilirubin, albumin and platelet count at 12 month
UK-PBC [124]	12	Baseline albumin and platelet count ALP, bilirubin and AST (or ALT) at 12 month

*UDCA* ursodeoxycholic acid, *ALP* alkaline phosphatase, *ULN* upper limit of normal, *AST* aspartate aminotransferase, *ALT* alanine aminotransferase

Recently, the two new continuous scoring systems, the GLOBE score [123] and the UK-PBC score [124], have been proposed based on multicenter studies with large number of patients included. GLOBE and UK-PBC scores are not only the response criteria but also important prognostic scores, which provided more accurate and individualized survival information than the existing models. These two scoring systems have recently been validated in a large international, multicenter PBC cohort, showing excellent prognostic performance [125].

Most studies demonstrated that ALP and total bilirubin are the two most important variables in evaluating UDCA response [126, 127]. Additionally, while most models assess biochemical response after 12 months of UDCA treatment, some reports suggest that evaluation after six months of UDCA treatment may have equivalent predictive utility [114, 118].

Prediction of response to UDCA is attempted even before the commencement of treatment. Based on two large-scale cohorts of PBC patients in the UK and Italy, Carbone et al. developed and validated a UDCA response score, consisting of bilirubin, ALP, transaminase, age, and lag time from diagnosis to treatment [128]. This score was also validated in a Japanese cohort [129]. Pretreatment prediction may help physicians identify patients with baseline characteristics conferring a high risk of incomplete response to UDCA and initiate a de novo combination of UDCA and another agent.

Recommendation:

4. It is recommended that evaluating all PBC patients for the biochemical response with appropriate criteria after 6 months of UDCA treatment (III, 1), or 12 months of UDCA treatment (II, 1)

### Second-line therapy for suboptimal responders to UDCA

**Fig. 4:**
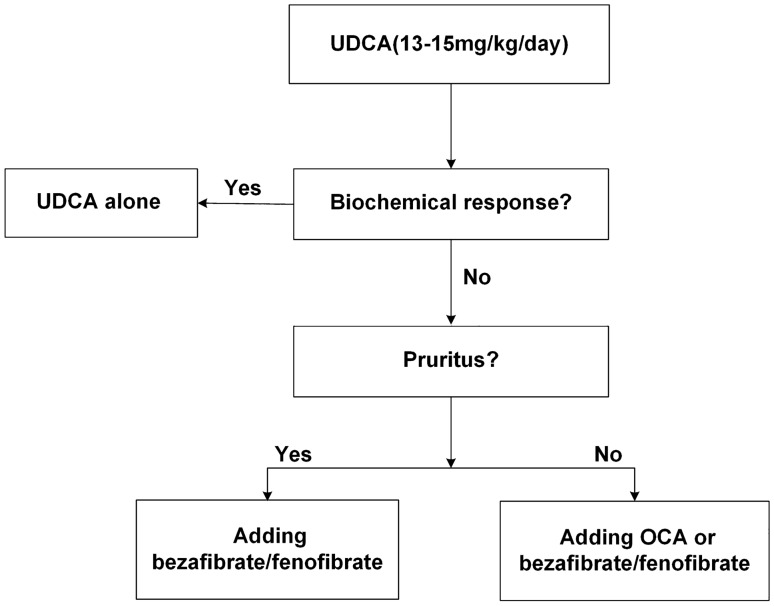
Risk-based approach for PBC patients. *UDCA* ursodeoxycholic acids, *OCA* Obeticholic acid

There is no consensus on therapies for patients with insufficient biochemical response to UDCA. In several clinical trials, obeticholic acid (OCA), fibrates, and budesonide proved to be effective and could be considered as second-line therapy for patients with insufficient UDCA response, but the long-term result needs further verification. Figure 4 showed the risk-based approach for managing PBC patients.

#### Second-line therapies approved in some regions of the world

##### Obeticholic acid

OCA has been approved as second-line therapy for PBC in the United States and Europe. OCA is a semi-synthetic hydrophobic bile acid analog that is highly selective for the farnesoid X receptor (FXR), a nuclear receptor abundantly expressed in the liver and enterocytes. In addition to directly regulating genes involved in the metabolism of bile acid synthesis, FXR signaling impacts inflammation, metabolic regulation, and liver fibrosis.

In a multicenter, double-blind phase II clinical trial, three doses of OCA (10, 25, and 50 mg/day) were added to UDCA for PBC patients with suboptimal response to UDCA (ALP > 1.5 × ULN). After three months, all three groups receiving OCA had more profound reductions in ALP level than the placebo group; moreover, in the 12-month open-label extension period, ALP levels continued to decrease [130]. In another randomized phase II clinical trial of OCA monotherapy in PBC patients who were UDCA-naïve or not taking UDCA for more than 3 months, ALP and other biochemical markers (GGT, ALT, conjugated bilirubin and IgM) were reduced in both OCA 10 mg and OCA 50 mg groups compared with the placebo group at the end of 3-month double-blinded phase, and the biochemical improvement was also observed at the end of 6-year open-label extention treatment [131].

In a double-blind phase III clinical trial from the PBC OCA International Study of Efficacy (POISE) group, after 12 months of OCA therapy (add-on to UDCA or as monotherapy) nearly half of the PBC patients who were prior biochemical non-responders or intolerance to UDCA achieved better biochemical improvement than the placebo group [132]. In a recent 3-year interim analysis from the 5-year open-label extension of the phase III POISE trial, ALP and total bilirubin were significantly decreased after 12 months and 48 months of OCA treatment compared to baseline [133].

Obeticholic acid was generally well tolerated after long-term follow-up, with pruritus (77%) and fatigue (33%) being the most common adverse events in phase III POISE trial [133]. Of note, the exacerbation of pruritus was dose-dependent, leading to treatment discontinuation in 15% (OCA 10 mg) to 38% (OCA 50 mg) of patients [131]. Moreover, patients treated with OCA exhibit a reduction of high-density cholesterol [131, 132]. It is still controversial if the reduction of high-density cholesterol increases the risk of cardiovascular events, although no serious adverse events were observed based on current results. In September 2017, the Food and Drug Administration of the United States released a warning that the use of OCA in PBC patients with decompensated cirrhosis (Child–Pugh-Turcotte B and C) was associated with clinical worsening or even death. Therefore, the use of OCA in PBC patients with decompensated cirrhosis was not recommended.

*Recommendation*:

5. It is recommended that OCA (starting at 5 mg/day, increasing to 10 mg/day after 6 months if tolerated well) be added to UDCA therapy for PBC patients (non-cirrhotic or cirrhosis with Child–Pugh–Turcotte A) and an inadequate response to UDCA or used as monotherapy in those intolerant to UDCA. Potential risks and adverse events of OCA should be discussed in detail with the patient, carefully evaluated and appropriately monitored. (I, 1)

#### Second-line therapies as off-label use

##### Fibrates (Fenofibrate and Bezafibrate)

Reports from the USA, Europe, and Asia demonstrated good efficacy of fenofibrate in PBC patients with suboptimal response to UDCA [134, 135]. Meta-analysis indicated that fenofibrate-UDCA combination therapy was more effective in decreasing ALP, GGT, IgM, and triglyceride levels than UDCA monotherapy, but it did not reduce ALT or improve pruritus [136]. Adverse events did not appear to increase in patients treated with fenofibrate-UDCA combination. However, possible liver and renal impairment in PBC patients, especially those with decompensated cirrhosis, need to be closely monitored [137].

Bezafibrate seems to have better efficacy than fenofibrate, but head-to-head comparison is still lacking. Studies showed that bezafibrate combined with UDCA could significantly decrease ALP, GGT, ALT, IgM, triglyceride, and total cholesterol levels [138, 139]. In 2018, a randomized, placebo-controlled phase III trial in PBC patients showed that bezafibrate combined with UDCA for 24 months achieved a higher rate of complete biochemical response than placebo plus UDCA in patients with incomplete response to UDCA monotherapy [140]. A recent Japanese study also reported that adding bezafibrate to UDCA in 118 PBC patients unresponsive to UDCA monotherapy resulted in improvements not only in liver biochemistry, UK-PBC, and GLOBE scores, but also in the long-term prognosis [141].

Additonally, recent studies showed that bezafibrate add-on treatment can completely or partially relief itching for PBC patients with suboptimal response to UDCA [139, 140]. Moreover, a phase III linical trial found a > 50% reduction of the intensity of pruritus score (VAS) in 55% PBC patients with moderate to severe pruritus after 21 days treatment of bezafibrate [142]. However, it has been reported that the bezafibrate-UDCA combination therapy was associated with more frequent adverse events than the UDCA monotherapy, including elevation of the serum creatinine levels and myalgia, polydipsia, aggravated pruritus, arthritis, leg edema, and gastrointestinal discomfort [138, 143].

*Recommendation*:

6. It is recommended that bezafibrate (400 mg/day) or fenofibrate (200 mg/day) could be added to UDCA for patients with an inadequate response to UDCA monotherapy. Adverse events should be closely monitored, especially in cirrhotic PBC patients. (I, 1).

##### Budesonide

Budesonide is the second generation of corticosteroids with high first-pass metabolism within the liver, therefore, it has fewer systemic side effects than conventional glucocorticosteroids. Two early reports of the multi-center prospective randomized study showed that adding budesonide (6–9 mg/day) to UDCA (15 mg/kg/day) in PBC patients exhibited better biochemical and histological improvement than continue UDCA monthotherapy [144, 145]. However, in a recent small scale randomized clinical trial, budesonide (9 mg/day) adding to UDCA (12 ~ 16 mg/kay/day) for 36 months achieve a better biochemical response but failed to achieve superior histological improvement in PBC patients with suboptimal response to UDCA monotherapy [146].

In summary, combination therapy with budesonide and UDCA might benefit non-cirrhotic PBC patients, but its efficacy on long-term clinical outcomes including mortality and requirement of liver transplantation still need further investigation. Of note is that significant increases in budesonide plasma levels were observed in late-stage PBC and were associated with severe side effects, including portal thrombosis. Therefore, the use of budesonide is not recommended for patients with cirrhosis [147].

*Recommendation*:

7. Budesonide (6–9 mg/day) might be added to non-cirrhotic PBC patients with suboptimal response to UDCA. (II, 2)

### Management of symptoms and complications

#### Pruritus

Pruritus is one of the characteristic symptoms of cholestasis and results in impaired health-related HRQoL in PBC patients. Although several candidate pruritogens, including lysophosphatidic acid, autotaxin, bile acids, bilirubin and endogenous opioids, have been proposed [148], the pathogenesis of pruritus has not been fully understood. A recent research found a novel bile acid receptor called MRGPRX4, which might be a promising therapeutic target for pruritus [149].

To date, cholestyramine is the first-line therapy for pruritus caused by cholestasis. The recommended dose is 4–16 g/day. The major side effects include abdominal bloating, constipation, and interference with the absorption of other drugs such as UDCA [150]. A four-hour interval is required between taking cholestyramine and other drugs.

If the patients are intolerant to cholestyramine, rifampicin could be the second choice. The recommended dose for rifampicin is 150 mg twice a day. For the patients without adequate response, the dose could be increased to 600 mg/day. Two meta-analysis indicated that rifampicin could effectively relieve itching caused by cholestasis [151, 152]. However, rifampicin could cause severe liver injury, hemolytic anemia, and renal damage and affect the metabolism and activity of other drugs [153–155]. Therefore, it is necessary to monitor the side effects of rifampicin.

Opioid antagonists could be considered as the third choice. Two randomized clinical trials and followed studies indicated that intravenous injection or oral administration of naloxone is effective for obstinate pruritus [151]. Naloxone should start from a low dose and slowly escalated to the optimal dose. The main adverse reactions were withdrawal symptoms. Nalfurafine hydrochloride, a selective kappa-opioid receptor agonis has been approved for intractable pruritus in patients with PBC in Japan [156]. As the 5-hydroxytryptamine system might be involved in the pathogenesis of pruritus, ondansetron and sertraline are also used to treat pruritus. To relieve severe or intractable pruritus, liver transplantation could be considered.

Currently, the efficacy and safety of linerixibat, a novel compound inhibiting ileal bile acid transporter (IBAT), is being evaluated for intracable pruritus in clinical trials of PBC patients. IBAT could diminish the total bile acid pool by inhibiting enter-hepatic circulaion, thereby reducing the pruritogens retended in bile acids. In the phase 2a study, 14 days of linerixibat treatment could reduce the pruritus severity [157], which has been confirmed by an international phase 2b trial [158]. It is generally well tolerated, although mild to moderate diarrhea occurred due to retention of bile acid in the colon.

*Recommendations*:

8. It is recommended that cholestyramine (4–16 g/day) as the first-line therapy for pruritus. To avoid interference with the absorption, take other medications including UDCA at least 1 h before or 4 to 6 h after taking cholestyramine. (II, 2)

9. Rifampicin (150–300 mg twice a day) can be used as second-line therapy for pruritus with close monitoring of side effects. (II, 2)

#### Vitamin D deficiency and osteoporosis

Previous studies reported that vitamin D deficiency was associated with the severity of chronic liver diseases [159]. EASL nutritional guidelines recommend supplement vitamin D orally in cirrhotic patients with vitamin D levels < 20 ng/ml, to reach serum vitamin D (25(OH)D) > 30 ng/ml [159]. For PBC patients, studies showed that vitamin D deficiency was common, especially in patients with more advanced disease and suboptimal response to UDCA therapy [160–162]. For perimenopausal and postmenopausal women, sufficient calcium (1000–1500 mg/day) and vitamin D (1000 IU/day) in the diet or as supplements are recommended if they have no history of renal stones [42].

The mechanisms for metabolic bone diseases (bone loss and osteoporosis) in PBC patients are complicated, involving the defect of absorption of fat-soluble vitamins and the direct effect of cholestasis on bone metabolism. The risk of fracture in PBC patients is two times higher than that in the healthy population. Supplements of calcium and vitamin D are recommended, with particular care in patients with a femur *T* score lower than − 1.5 [163].

A meta-analysis of six randomized clinical trials provided insufficient evidence to claim or refute a benefit for bisphosphonates (alendronate, etidronate, ibandronate) in treating PBC-related osteoporosis [164]. However, a recent randomized clinical trial showed that either weekly alendronate or monthly ibandronate treatment could improve bone mass in patients with PBC [165].

Bisphosphonates are generally well-tolerated, and the potential side effects include gastroesophageal irritation, osteonecrosis of the jaw, musculoskeletal pain, and atrial fibrillation. Bisphosphonates are not recommended for people with severe impairment of renal function or hypocalcemia. People with specific problems of the esophagus may not be able to take the oral tablets.

So far, hormone replacement therapy in women with PBC is not supported by reliable evidence [166]. Recently, a report comfirmed the efficacy and safety of denosumab, a fully human monoclonal antibody against the receptor activator of nuclear factor-kappaB ligand (RANKL), for osteoporosis in patients with PBC [167].

*Recommendations*:

10. It is recommended that all PBC patients should be evaluated for serum vitamin D status. (II, 2)

11. It is recommended that all PBC patients should be evaluated for osteoporosis, especially in postmenopausal women. (III, 2)

12. Patients should intake enough calcium (1000–1500 mg/day) and vitamin D (1000 IU/day) in the diet or as supplements if needed, according to local practice. (III, 2)

13. Bisphosphonates (alendronate 70 mg weekly or ibandronate 150 mg monthly or others) can be considered in patients with osteoporosis. Bisphosphonates should be used with caution in patients with esophageal varices, and the side effects should be monitored in all patients. (III, 2)

14. Data on denosumab efficacy in PBC patients with osteoporosis is very limited in Asia Pacific region, therefore, a clear recommendation cannot be made (or supported). (III, 2)

#### Fatigue

No effective therapy for fatigue is available at this moment. Though multiple candidates have been tested, such as UDCA, fluoxetine, colchicine, methotrexate, ondansetron and S-ademetionine, only modafinil exhibits promising results. An observational study indicated that modafinil could attenuate fatigue in PBC patients caused by excessive daytime sleepiness and improve the Epworth Sleepiness Scale and PBC-40-Quality of Life [168].

The side effects of modafinil include insomnia, nausea, headache, and nervousness. More evidence is required to verify the efficacy of modafinil. The physicians should also pay attention to other factors associated with fatigue, such as anemia, hypothyroidism, depression, and sleep disorders.

*Recommendation*:

15. No specific medical therapy is available for fatigue. Treating co-existent conditions such as anemia, extra-hepatic autoimmune disease, sleep disturbance, and depression are recommended to manage fatigue. (III, 2)

#### Portal hypertension

Patients with PBC progress into portal hypertension as a result of biliary cirrhosis. A screening esophagogastroduodenoscopy (EGD) should be performed in patients with cirrhotic features at the time of the diagnosis. However, esophageal varices can develop early in the disease course, even before the establishment of cirrhosis [169]. Nodular regenerative hyperplasia may play a role in portal hypertension development of early-stage PBC patients [66]. The Baveno-VI criteria (LSM by TE < 20 kPa and platelet count > 150 × 10^9^/L) can be used to identify patients who may not need screening EGD. One study showed that this strategy could avoid 39% of screening EGD with a false negative rate of 0% [170]. Nonselective beta-blockers and/or endoscopic band ligation is indicated in patients with large esophageal varices or variceal hemorrhage.

*Recommendation*:

16. Patients with features of portal hypertention (ie. splenomegaly, thrombocytopenia) should be screened for gastroesophageal varices. (II, 2)

### Hepatocellular carcinoma

The reported incidences of PBC-related HCC range from 2.4 to 6.6 cases per 1000 patient-years, which is two times higher in males than that in females [67, 68, 171–173]. A recent meta-analysis showed that the PBC-related HCC incidence was 5.77 per 1000 person-years in Asia, which was similar to that in North America (5.10 per 1000 person-years), but higher than that in Europe(2.67 per 1000 person-years) [174].

As reported, male sex and advanced histological stage independently associated with the development of HCC [67, 68, 171–173]. In addition, an international cohort study showed that biochemical non-response at one year of UDCA treatment (Paris-II not fulfilled) significantly increased the future risk of HCC [172]. Other risk factors associated with HCC in PBC including older age, any signs of portal hypertension, thrombocytopenia, past HBV infection, diabetes, obesity and alcohol consumption, as summurized by two recent reviews [67, 69].

Taken together, close monitoring for HCC development is strongly recommended for high-risk patients with PBC, such as males, patients with advanced-stage disease, and non-responders to UDCA.

*Recommendation*:

17. Close monitoring of HCC is strongly recommended for males, patients with advanced-stage disease, and non-responders to UDCA. (II, 2)

### Liver transplantation

Liver transplantation (LT) should be considered in PBC patients who have progressed to decompensated cirrhosis (ascites, variceal hemorrhage, or hepatic encephalopathy), with a model for end-stage liver disease (MELD) score > 15, or with a Mayo Risk Score > 7.8 [43, 175, 176]. Severe intractable pruritus that heavily compromises the HRQoL is an exceptional indication for transplantation.

The long-term post-tranplant survival is relatively optimistic in PBC patients, with a 5-year survival rate of 80–85% [177–179]. The 5-, 10-, and 15- year post-transplant recurrence occurs approximately in 22%, 21–37%, and 40% of liver allografts, respectively, with a median time range of 3–6.9 years [180, 181]. The diagnosis of PBC recurrence is based on histological features (granulomatous cholangitis and/or florid duct lesions) and biochemical abnormalities [178, 182], since AMA may remain positive after LT even without PBC recurrence. Younger age, use of tacrolimus, and biochemical cholestasis after LT were related to PBC recurrence [180].

Previous studies reported that PBC recurrence did not significantly compromise the long-term outcomes. However, a recent large-scale retrospective cohort study demonstrated that PBC recurrence significantly compromised graft and patient survival rates [180]. Prophylactic use of UDCA is safe and effective in preventing PBC recurrence after liver transplantation [183].

*Recommendations*:

18. It is recommended that liver transplantation should be considered in patients with decompensated cirrhosis, MELD ≥ 15, Mayo Risk Score > 7.8, or severe, intractable pruritus. (II, 1)

19. Post-transplant UDCA treatment is safe and effective in improving liver function tests and prevent PBC recurrence. (II, 1)

## Special conditions

### PBC with AIH features (formerly known as PBC-AIH overlap syndrome)

PBC and AIH are nosological entities characterized by different histological and serological phenotypes. They can coexist in the same patients with either a sequential or a simultaneous presentation. As the most common overlap form in autoimmune liver diseases, the prevalence of PBC with AIH features is approximately 5 ~ 15% of all PBC patients [184, 185]. Similarly, AMA can also be detected in 5–35% of patients with well-established AIH [71]; whether these patients will develop typical PBC is till to be defined [186].

The explicit clinical or pathological definition of PBC with AIH features is still lacking, although the “Paris Criteria” (1998) are frequently used in clinical practice [187]. According to these criteria, to diagnose the PBC with AIH features, the patients must meet at least two of each three criteria of PBC and AIH. For PBC: (1) serum ALP levels at least two times ULN or serum GGT levels at least five times ULN; (2) the presence of AMA and/or AMA-M2; (3) a liver biopsy showing florid duct lesions. For AIH: (1) ALT levels at least five times ULN; (2) serum IgG levels at least two times ULN or the presence of anti-smooth muscle antibody (ASMA); (3) a liver biopsy showing moderate/severe interface hepatitis (mandatory).

However, ASMA positivity is less frequent, and the serum IgG levels are seldom above 2 × ULN, especially in the Asia-Pacific region [188, 189]. Thus, the “Paris Criteria” are probably too stringent for diagnosing the PBC with AIH features in this region. A study from China showed that the serum IgG levels ≥ 1.3 × ULN had a 60% sensitivity and a 97% specificity for PBC with AIH features, which is more sensitive than “Paris Criteria” (IgG levels ≥ 2 × ULN) [190].

Autoantibodies profile were also explored as potential diagnostic markers for PBC with AIH features. Muratori et al. found that stimultaneous positivity for AMA and anti-dsDNA had a 98% specificity for diagnosis of PBC with AIH features [191]. In line with this, a recent study demonstrated that anti-dsDNA could be the diagnostic marker of PBC with AIH features, whereras other autoantibodies including anti-p53, Ro52/TRIM21, anti-KLHL-12 and anti-HK-1 were not significantly associated with PBC with AIH features [192].

Of note, authors from Europe and the US strongly discourage using the AIH scoring system (International Autoimmune Hepatitis Group; IAIHG 1999) or the simplied score (IAIHG 2008) for diagnosing PBC with AIH features [184, 193–195].

Patients with PBC with AIH features have poorer outcomes than those with AIH or PBC alone [196]. A nation-wide study from Japan suggested that the simplified AIH scoring system (IAIHG 2008) was beneficial for selecting patients who require corticosteroids administration [197]. For these patients, two therapeutic approaches could be considered. One is to treat the patients with UDCA for 3–6 months, and add immusuppresive therapy if the levels of ALT/AST and IgG are still not improved. Another approach is to start UDCA and immusuppresive therapy simultaneously if the evidence for PBC with AIH features is strong. Studies showed that corticosteroids with or without azathioprine, or second-line immunosuppressants (i.e., mycophenolate mofetil, tacrolimus, and cyclosporine A) added to UDCA is useful to increase the response rates and improve the prognosis [198, 199].

*Recommendations*:

20. The diagnosis of PBC with AIH features could be made in PBC patients if two of the three following criteria are met: (1) moderate/severe interface hepatitis in liver histology (mandatory); (2) serum ALT/AST more than 5 times ULN; and (3) IgG level more than 1.3 times ULN or the presence of ASMA. (III, 2)

21. mmunosuppressive agents (including corticosteroid with or without azathioprine or mycophenolate mofetil) could be used as add-on therapy to UDCA, or de novo combination therapy with UDCA. (III, 2)

### AMA-negative PBC

About 5% of PBC patients are negative for AMA [70, 71]. AMA-negative PBC patients tend to have lower IgM levels and higher titers of PBC-specific ANA (anti-gp210 and/or anti-sp100) than AMA-positive PBC patients [200]. Most studies suggested that the clinical presentation, liver histology and clinical course of AMA-negative PBC were nearly identical to AMA-positive PBC [200, 201]. However, some studies indicated that AMA-negative PBC was associated with more severe bile duct damage on histology and worse clinical outcomes [202]. [203].

Therefore, to avoid undue delay of the treatment, liver biopsy is recommended for this kind of patients to confirm the diagnosis of AMA-negative PBC and to exclude the coexistence of AIH or NASH.

*Recommendations*:

22. It is recommended that liver biopsy should be performed on patients who present otherwise unexplained cholestatic liver biochemistry and negative for AMA, anti-gp210, or anti-sp100 to confirm the diagnosis of PBC. (III, 2)

### Isolated AMA positivity

Studies demonstrated that some individuals with AMA positivity and normal serum ALP levels had shown no clinical evidence of PBC [204–206]. Dahlqvist et al. reported that only 1 out of 6 AMA-positive patients with normal serum ALP levels would progress to PBC during follow-up for five years [205]. Gulamhusein et al. also found that none of the first-degree relatives of PBC patients who were AMA-positive and with normal ALP at baseline developed PBC during follow-up [206]. In line with this finding, several other studies showed that the prevalence of AMA positivity in healthy subjects was higher than the prevalence of PBC [207–209].

However, a recent single-center study from China showed that more than 80% of patients with normal ALP and positive AMA had histological evidence of PBC [210]. Similar result was also reported from a multi-center Swiss study [211]. Histologically proven PBC patients who had AMA positivity and with normal ALP had significantly higher ELISA-determined AMA titers, higher level of ALP (within normal range), and elevated IgM than individuals with positive AMA only [210]. Therefore, regular following-up and timely liver biopsy are recommended for these patients since prompt initiation of UDCA therapy may be beneficial.

*Recommendation*:

23. AMA reactivity alone is not sufficient to diagnose PBC. AMA-positive patients with normal serum liver tests should be followed up with an annual biochemical reassessment for the presence of liver disease. A liver biopsy may be considered in selected patients (eg. patients with elevated IgM, high titer of AMA, or ALP close to ULN) to identify preclinical PBC. (III, 2)

### Pregnancy

Most studies reported that maternal and fetal outcomes were good for pregnant women with PBC [212–214]. However, cirrhotic PBC patients have a higher risk of maternal and fetal complications, therefore, they may need special counseling.

UDCA is widely used in patients with intrahepatic cholestasis of pregnancy and is presumed to be safe during the second and third trimester [215–217]. Studies did not show any unexpected side effects in pregnant women with PBC or PSC who received UDCA during the first trimester [213, 214, 218]. However, information on this issue still remains too scarce to give a specific recommendation.

There is also a paucity of data on the safety of UDCA during breastfeeding. Rudi et al. firstly reported that treatment of UDCA 750 mg/day did not result in appearance of UDCA in the breast milk in a PBC patient at breastfeeding [219]. In another case [220], UDCA treatment was initiated at 7.5 mg/kg/day and gradually increased to 25 mg/kg/day, with no effects on the bile acid content in the breast milk. In light of published cases there are no severe side effects among babies whose mothers continued UDCA during breastfeeding [213]. These results suggested that it appears safe to receive UDCA during breastfeeding.

*Recommendations*:

24. Pregnancy can be advised in PBC patients at childbearing age. Patients with features of cirrhosis should be well informed about the possible maternal and fetal complications. (III, 2)

25. Although data on UDCA treatment during pregnancy and breastfeeding remains limited, continued use of UDCA can be considered in those patients after special counseling on these particular issues. (III, 2)

## Natural history and prognosis

### Natural history and clinical course

The natural history of PBC can be divided into four phases [221]: preclinical, asymptomatic, symptomatic, and terminal phase. In the pre-UDCA era, PBC patients were usually diagnosed at an advanced stage due to the absence of screening liver chemistries, limited availability of AMA tests and effective therapy, with a median survival of 6–10 years [222–225].

UDCA treatment has dramatically improvd the prognosis of PBC patients. The survival rate of the early-stage patients with complete response to UDCA therapy was similar to that of the general population [111, 115]. Liver transplant-free survival rate was significantly improved even in those with incomplete response to UDCA compared with no treatment [109]. In the UDCA era, the 5-year liver-related fatality and decompensation incidence in the Asia–Pacific region were 4.02% and 6.95%, respectively [4], which were comparable to that in the Western countries.

### Risk factors for poor clinical outcomes

In addition to biochemical response to UDCA, several clinical, biochemical, and histological features also have prognostic significance for PBC patients. Cirrhosis at baseline and higher bilirubin are widely recongnized as strong predictors of worse long-term outcomes. Younger age at presentation [112, 226], alcohol consumption, smoking [96], and the onset of symptoms [227, 228] are also predictors of poorer prognosis. It is controversial whether male sex is associated with poorer long-term outcomes in PBC [45, 112, 226]. It is reported that anti-gp210 and anti-sp100 antibodies are associated with the advanced course and poorer prognosis [84, 229–232]. Additionally, histological features including fibrosis or cirrhosis, interface activity, ductopenia, and chronic cholestasis are critical facotors to predict biochemical response and clinical outcome in PBC patients [111, 112, 119, 233–235].

Finally, the impact of past HBV infection on the clinical outcomes of PBC is controversial. One study found that past HBV infection was a risk factor for HCC occurrence in PBC patients [67]. However, another large cohort recently reported from China demonstrated that past HBV infection (HBsAg negative and anti-HBc positive) did not compromise the prognosis of PBC patients [45].

Overall, baseline disease stage and biochemical response to UDCA are the two most important predictors for PBC patients' survival. Risk stratification based on clinical, biochemical, and histological features of PBC patients will facilitate the optimization of clinical management.

## Future research and horizons

Identification of mitochondrial autoantigens have facilitated the earlier diagnosis and introduction of UDCA as first-line therapy significantly has altered the natural history of PBC. Nevertheless, many mysteries and unmet medical needs still exist in the understanding, diagnosis, and management of PBC.

Firstly, although PBC is considered an autoimmune liver disease, it remains unclear what environmental factors directly trigger the loss of self-tolerance to BECs, thereby leading to injury of intrahepatic ductules. While diagnostic utility of AMAs in PBC is remarkable, its pathogenic role remains to elucidate. Therefore, experimental studies with animal models recapitulating human PBC and relevant translational studies would generate new knowledge on the etiopathogenesis, thereby facilitating the discovery of novel therapeutic modalities.

Secondly, the clinical features and natural history of atypical clinical phenotype (such as preclinical or subclinical, AMA-negative disease, and vanishing bile duct) or variants of PBC (PBC with AIH features, PBC recurrence after liver transplantation) should be further investigated to optimize the management strategy for these subgroups.

Thirdly, although UDCA is the treatment of choice for PBC, the therapeutic responses are not always predictable. Hence, it is necessary to investigate the clinical and laboratory stratification factors to provide individualized care with available therapeutic agents, including UDCA, OCA, fibrates, and budesonide.

Finally, collaborative efforts of scientists, clinicians, methodologists, and ethical experts are pivotal to accelerate the clinical development of novel therapies. The potential targets include the critical molecular pathways that may trigger the autoimmune reactivity against intrahepatic BECs or mediate disabling symptoms such as intractable fatigue and pruritus. Specifically, the merit of the early use of immunomodulatory therapy to change the natural history of PBC should be explored in the near future. Theoretically, immunological agents such as rituximab (anti-CD20 antibody) and ustekinumab (anti-IL-12/IL-23 antibody) are likely efficacious for patients with early-stage PBC, but not for patients at an advanced stage with UDCA-resistance. Obviously, the stringent ethical rules should be leveraged in the context of enormous unmet clinical needs to facilitate the design and conduct of clinical trials of novel therapies for PBC.

## Data Availability

Not applicable.
